# Enhancing Palliative Care for Patients With Advanced Heart Failure Through Simple Prognostication Tools: A Comparison of the Surprise Question, the Number of Previous Heart Failure Hospitalizations, and the Seattle Heart Failure Model for Predicting 1-Year Survival

**DOI:** 10.3389/fcvm.2022.836237

**Published:** 2022-04-11

**Authors:** Moritz Blum, Laura P. Gelfman, Karen McKendrick, Sean P. Pinney, Nathan E. Goldstein

**Affiliations:** ^1^Department of Internal Medicine and Cardiology, Charité – Universitätsmedizin Berlin, Berlin, Germany; ^2^Brookdale Department of Geriatrics and Palliative Medicine, Icahn School of Medicine at Mount Sinai, New York, NY, United States; ^3^James J. Peters Veterans Affairs Medical Center, Geriatric Research Education and Clinical Center, Bronx, NY, United States; ^4^Department of Medicine, University of Chicago Medicine, Chicago, IL, United States

**Keywords:** survival prediction, number of hospitalizations, Seattle Heart Failure Model, advanced heart failure, surprise question, palliative care

## Abstract

**Background:**

Score-based survival prediction in patients with advanced heart failure (HF) is complicated. Easy-to-use prognostication tools could inform clinical decision-making and palliative care delivery.

**Objective:**

To compare the prognostic utility of the Seattle HF model (SHFM), the surprise question (SQ), and the number of HF hospitalizations (NoH) within the last 12 months for predicting 1-year survival in patients with advanced HF.

**Methods:**

We retrospectively analyzed data from a cluster-randomized controlled trial of advanced HF patients, predominantly with reduced ejection fraction. Primary outcome was the prognostic discrimination of SHFM, SQ (“Would you be surprised if this patient were to die within 1 year?”) answered by HF cardiologists, and NoH, assessed by receiver operating characteristic (ROC) curve analysis. Optimal cut-offs were calculated using Youden’s index (SHFM: <86% predicted 1-year survival; NoH ≥ 2).

**Results:**

Of 535 subjects, 82 (15.3%) had died after 1-year of follow-up. SHFM, SQ, and NoH yielded a similar area under the ROC curve [SHFM: 0.65 (0.60–0.71 95% CI); SQ: 0.58 (0.54–0.63 95% CI); NoH: 0.56 (0.50–0.62 95% CI)] and similar sensitivity [SHFM: 0.76 (0.65–0.84 95% CI); SQ: 0.84 (0.74–0.91 95% CI); NoH: 0.56 (0.45–0.67 95% CI)]. As compared to SHFM, SQ had lower specificity [SQ: 0.33 (0.28–0.37 95% CI) vs. SHFM: 0.55 (0.50–0.60 95% CI)] while NoH had similar specificity [0.56 (0.51–0.61 95% CI)]. SQ combined with NoH showed significantly higher specificity [0.68 (0.64–0.73 95% CI)].

**Conclusion:**

SQ and NoH yielded comparable utility to SHFM for 1-year survival prediction among advanced HF patients, are easy-to-use and could inform bedside decision-making.

## Introduction

Heart Failure (HF) is a life-limiting condition which usually progresses to an advanced stage with debilitating symptoms, emotional burden, and reduced quality-of-life (QOL) (1). Accordingly, current guidelines promote the use of palliative care for patients with advanced HF (2). Palliative care is an interdisciplinary approach aimed to alleviate physical and psychological symptoms and restore and maintain QOL in patients with serious illness and several studies demonstrated its benefit in HF patients (3–5). Palliative care is not limited to end-of-life care and should be integrated early in the disease course for patients with unmet symptoms and needs (3).

Provision of palliative care is not contingent upon a high probability of dying, and should be administered based on clinical necessity. However, clinicians often do not refer patients with advanced heart failure to palliative care until very late in their disease trajectory (6), and better information about prognosis may raise awareness about patients’ clinical needs for palliative care. Also, prognostication plays an important role in palliative medicine, in particular when it comes to timing and planning of discharge, referral to hospice, and transition to end-of-life care, as well as to addressing patients’ and caregivers’ questions and uncertainties (7, 8). Estimates of life expectancy are particularly critical for decision making regarding advanced treatment options for cardiac conditions, such as ICD implantation and transcatheter aortic valve implantation, for which recent guidelines require a minimum life expectancy of 1 year (2).

To date, survival prediction has mostly been approached by means of sophisticated multivariable scores, such as the Seattle Heart Failure Model (SHFM) (2). Although these scores provide fair prognostic discrimination in patients with advanced HF, their utility in clinical practice is limited because they require a large amount of variables some of which are not routinely collected (e.g., lymphocyte count and uric acid) and the use of dedicated calculators.

Simpler prognostic approaches could facilitate the widespread use of survival prediction. The surprise question (SQ), which asks clinicians “Would you be surprised if this patient died in the next 12 months?,” and the number of HF hospitalizations within the last 12 months (NoH) are promising prognostic tools which can be easily applied at the bedside by tapping into clinician intuition and basic history-taking. In previous studies of HF patients, both metrics were found to be closely associated with mortality (9–11).

## Objectives

The aim of this study was to compare the discrimination of the SHFM, the SQ, the NoH and the combination of SQ and NoH for prediction of 1-year survival in a population of advanced HF patients.

## Methods

This study was a retrospective, secondary analysis of advanced HF patients with an implantable cardiac defibrillator (ICD) enrolled in the Working to Improve diScussions about DefibrillatOr Management (WISDOM) trial (NCT01459744, NCT01454817). The WISDOM trial was a multisite, single-blinded, cluster-randomized controlled trial to test whether a clinician-centered intervention of educational content and automated reminders increased the likelihood of ICD deactivation conversations and it is described in detail elsewhere (12).

The final inclusion criteria encompassed inpatients, as well as outpatients with advanced HF, an implanted ICD and a high risk of dying according to the following criteria: for inpatients, at least one other HF hospitalization within the last 12 months, or two out of four objective measures (age >70 years, blood urea nitrogen >43 mg/dL, serum creatinine >2.75 mg/dL, systolic blood pressure <115 mmHg) were required; for outpatients, at least two HF hospitalizations within the last 12 months, or New York Heart Association (NYHA) class IV dyspnea, or NYHA class III dyspnea and either at least one HF hospitalization within the last 12 months or two out of four objective measures (age >70 years, blood urea nitrogen >43 mg/dL, serum creatinine >2.75 mg/dL, systolic blood pressure <115 mmHg) were required. The detailed inclusion and exclusion criteria are published separately (13).

For the present study, we included all patients with complete data on SHFM, SQ, NoH and 1-year survival status, which was the outcome of interest. SHFM-predicted survival was calculated from baseline variables (14). Clinical research coordinators collected clinical variables at baseline and ascertained survival status during follow-up. The SQ was answered by attending HF physicians who were board certified in Advanced HF and Transplant Cardiology, Cardiology and Internal Medicine which would require a minimum of approximately 7 years of clinical experience. The NoH was abstracted manually from electronic medical records.

Statistical analysis was performed using Stata Statistical Software: Release 16 (StataCorp LLC, College Station, TX, United States). Categorical variables are presented as absolute number and percentage. Numerical variables are presented as mean ± standard deviation. Baseline variables were compared using independent *t*-test or Chi-square test, whichever was applicable. Discrimination of the SHFM, SQ, and the NoH for 1-year survival status was assessed by means of receiver operating characteristic (ROC) curve analysis. For the SHFM and the NoH, we empirically determined a cut-off based on Youden’s index (15). We compared discrimination for prediction of 1-year survival status of the SHFM, the SQ and the NoH based on area under the ROC curve (AUC), sensitivity, specificity, positive predictive value (PPV), and negative predictive value (NPV). To allow for statistical inference regarding the comparison of different prognostic approaches, 95% confidence intervals (95% CI) were computed.

## Results

In our sample of 535 patients, 82 (15.3%) had died at 1 year. Population characteristics are detailed in Table 1. The mean age was 61.5 ± 13.9 years, 379 patients (70.8%) were male and 286 (52.4%) were White. Overall, the mean left ventricular ejection fraction (LVEF) was 24.3 ± 10.1%. The vast majority of patients [485 (94.9%)] had a reduced LVEF, a minority 10 (2.0%) had mid-range EF, and 16 (3.1%) had preserved LVEF. The average predicted 1-year survival according to the SHFM model at baseline was 86.8% ± 24.9. The SQ was answered with “No, I would not be surprised if this patient was to die within 1 year” in 375 (70.1%) of patients overall. And 246 patients (46.0%) were found to have an NoH ≥ 2 at baseline.

**TABLE 1:** Baseline characteristics of the study population overall and stratified for survival status after 1-year follow-up.

	Overall (*N* = 535)	Died (*N* = 82)	Survived (*N* = 453)	*p*-value
Gender, male	379 (70.8)	57 (69.5)	322 (71.1)	0.77
Age – years	61.5 ± 13.9	62.3 ± 13.9	61.4 ± 13.9	0.57
Race/ethnicity*				0.18
White	286 (54.2)	49 (59.8)	237 (53.1)	
Black	213 (40.3)	31 (37.8)	182 (40.8)	
Hispanic	74 (13.9)	10 (12.2)	64 (14.2)	
Other	17 (3.2)	0 (0.0)	17 (3.2)	
LVEF – %	24.3 ± 10.1	22.5 ± 9.0	24.6 ± 10.3	0.10
HF classification**				0.229
HFrEF	485 (94.9)	77 (98.7)	408 (94.2)	
HFmrEF	10 (2.0)	0 (0.0)	10 (2.3)	
HFpEF	16 (3.1)	1 (1.3)	15 (3.5)	
NYHA class				<0.001
I	3 (0.6)	0 (0)	3 (0.7)	
II	43 (8.0)	5 (6.1)	38 (8.4)	
III	412 (77.0)	52 (63.4)	360 (79.5)	
IV	77 (14.4)	25 (30.5)	52 (11.5)	
Ischemic etiology	235 (44.0)	38 (46.3)	197 (43.6)	0.64
VAD/Tx candidate	256 (47.9)	36 (43.9)	220 (48.6)	0.44
**Prediction variables**				
Surprise question***				0.003
No	375 (70.1)	69 (84.1)	306 (67.5)	
Yes	160 (29.9)	13 (15.9)	147 (32.5)	
Number of HF hospitalizations****				0.046
≥2	246 (46.0)	46 (56.1)	200 (44.2)	
<2	289 (54.0)	36 (43.9)	253 (55.8)	
SHFM predicted 1-year survival				<0.001
<86%	266 (49.7)	62 (75.6)	204 (45.0)	
≥86%	269 (50.3)	20 (24.4)	249 (55.0)	

Continuous variables are shown as mean ± standard deviation. Categorical variables are shown as number (percentage). HF, heart failure; LVEF, left ventricular ejection fraction; NYHA, New York Heart Association; SHFM, Seattle Heart Failure Model; Tx, transplantation; VAD, ventricular assist device. *Not mutually exclusive. **HF classification according to the Universal Definition and Classification of Heart Failure (27). ***Yes = would be surprised if patient died within 1 year; No = would not be surprised if patient died within 1 year. ****Within the last 12 months before enrollment.

For the SHFM and the NoH, optimal cut-offs based on ROC curve analysis were found to be a predicted survival <86% and ≥2 hospitalizations, respectively. Prognostic performance metrics of the SHFM, the SQ, and the NoH are shown in Table 2. The SHFM yielded an area under the ROC curve (AUC) of 0.65 (0.60–0.71 95% CI), a sensitivity of 0.76 (0.65–0.84 95% CI), and a specificity of 0.55 (0.50–0.60 95% CI). The SQ demonstrated a comparable AUC [0.58 (0.54–0.63 95% CI)], similar sensitivity [0.84 (0.74–0.91 95% CI)], but significantly lower specificity [0.33 (0.28–0.37 95% CI)] compared to the SHFM. The NoH demonstrated a comparable AUC [0.56 (0.50–0.62 95% CI)], similar sensitivity [0.56 (0.45–0.67 95% CI)], and similar specificity [0.56 (0.51–0.61 95% CI)] compared to the SHFM. The combination of a positive a SQ and a NoH ≥ 2 showed similar a similar AUC [0.60 (0.54–0.66 95% CI)], lower sensitivity [0.51 (0.40–0.62 95% CI)], but significantly higher specificity [0.68 (0.64–0.73 95% CI)] compared to the SHFM. Of note, the PPV and NPV did not differ significantly between any of the prognostic approaches, with PPVs and NPVs ranging consistently around 20 and 90%, respectively (Table 2).

**TABLE 2:** Discrimination of the Seattle Heart Failure Model, the surprise question, the number of hospitalizations and a combination of the latter for prediction of 1-year survival.

	Seattle Heart Failure Model	Surprise question	No. of hospitalizations	Surprise question + No. of hospitalizations
Cut-off	Predicted survival <86%	“No, would not be surprised if patient died.”	NoH ≥ 2	“No, would not be surprised” **+** NoH ≥ 2
AUC	0.65 (0.60, 0.71)	0.58 (0.54, 0.63)	0.56 (0.50, 0.62)	0.60 (0.54, 0.66)
Sensitivity	0.76 (0.65, 0.84)	0.84 (0.74, 0.91)	0.56 (0.45, 0.67)	0.51 (0.40, 0.62)
Specificity	0.55 (0.50, 0.60)	0.33 (0.28, 0.37)	0.56 (0.51, 0.61)	0.68 (0.64, 0.73)
PPV	0.23 (0.18, 0.29)	0.18 (0.15, 0.23)	0.19 (0.14, 0.24)	0.23 (0.17, 0.30)
NPV	0.93 (0.89, 0.95)	0.92 (0.87, 0.96)	0.88 (0.83, 0.91)	0.89 (0.85, 0.92)

*Outcome of interest was survival status at 1-year follow up. AUC, area under the receiver operating characteristic curve; NoH, number of heart failure hospitalizations within the last 12 months; NPV, negative predictive value; PPV, positive predictive value; SQ, surprise question; SHFM, Seattle Heart Failure Model.*

## Discussion

In a sample of advanced HF patients at a high risk of dying and of which the vast majority had reduced ejection fraction, two simple single-measure bedside tools, the SQ, the NoH, and the combination of the SQ and NoH performed as well as the complex multivariable SHFM for prediction of 1-year survival status in patients with advanced HF.

The prognostic utility of the SHFM in our study as assessed by the AUC [0.65 (0.60–0.71 95% CI)] was comparable to previous studies of advanced HF populations which reported AUCs ranging from 0.63 to 0.76 (16–18). Three previous studies assessed the SQ in HF populations: Aaronson et al. (19) studied 199 patients presenting to the emergency room with acute HF and found an AUC of 0.68 which was significantly higher than what we found [AUC 0.58 (0.54–0.63 95% CI)]. Straw et al. (9) studied 129 patients hospitalized with acute HF and found a sensitivity of 0.88, which was comparable to our findings [sensitivity 0.84 (0.74–0.91 95% CI)], and a specificity of 0.59, which was significantly higher than in our study [specificity 0.33 (0.28–0.37 95% CI)]. Gonzalez-Jaramillo et al. (10) studied 174 patients recruited from an HF clinic and found a sensitivity of 0.85 (0.69–1.00 95% CI), and a specificity of 0.57 (0.49–0.65 95% CI). Overall, our findings are in line with previous research. Of note, inclusion criteria and event rates varied between those and the present study which limits comparability.

The SHFM is one of the most widely known prognostic models for survival prediction in HF patients, demonstrating robust discrimination in different advanced HF populations and generally considered a gold standard for both clinical practice and research purposes (16–18). It also allows clinicians to estimate the effect of medication and devices such as ICD on mortality (14). For score calculation, users have to access an online application via computer or smartphone (20). The SHFM requires measures of 14 variables including the lymphocyte count and uric acid, which are usually not part of the standard HF laboratory work-up (20). Those requirements severely hinder widespread clinical use of this prognostic tool. Unlike the SHFM, the SQ and the NoH can be easily implemented as part of the routine clinical work-up of advanced HF patients by leveraging clinician intuition and basic history-taking. In our sample, we demonstrated that these simple prediction tools provided similar prognostic discrimination compared to the SHFM. Therefore, we believe that the SQ and the NoH are valuable additions to the “prognostic tool-box” of HF specialists as well as palliative care clinicians, either as quick bedside alternatives, or complementary to more sophisticated approaches.

Previous studies have repeatedly framed the SQ a possible trigger for palliative care referral (19, 21, 22). However, current guidelines and position statements clearly state that palliative care utilization should be based on unmet physical, psychological, social and spiritual needs and poor QOL instead of estimated prognosis (1–3). Yet, wide-spread use of evidence-based prognostication, facilitated by simple tools such as the SQ and the NoH, could inform a variety of clinical scenarios and decisions: Estimation of a patient’s prognosis is important for improving patients’ understanding of their illness trajectory, which remains poor (23), initiating discussions around goals of care and treatment preferences at the end-of-life (24), referring to hospice, which is underused in advanced HF (25), and engaging in decisions regarding the implantation, continuation or discontinuation of device therapies such as ICD or left ventricular assist devices. The goal of our work is not to imply that referral to palliative care should be based on prognosis; instead we hypothesize that the use of simple tools to determine prognosis might nudge clinicians toward considering palliative care referrals for patients with unmet needs earlier in their illness trajectory. Given the fact that patients with advanced HF are referred to palliative care late (6, 26), we believe that easier tools to flag severely ill patients might create a path to earlier integration of supportive and palliative services into the patient’s overall plan of care.

Of note, all prediction tools – and the SQ in particular – are flawed by low specificity translating into a poor positive predictive value. This tendency toward underestimating survival and the generally low accuracy of less than 0.7 AUC for all prognostic approaches under investigation must be kept in mind when applying these tools in clinical practice. Our findings have to be interpreted with caution. As demonstrated previously, the reliability of the SQ depends on training and clinical experience. In our study, the SQ was answered by specialized HF cardiologists – discrimination might be worse when used by other clinicians. Although the inclusion criteria were intended to select patients at high risk of dying, 1-year mortality in our study population was surprisingly low. Low event rates might limit statistical power to detect statistically significant differences. Furthermore, our sample had a rather young mean age, and a high proportion of patients who were candidates for transplantation or VAD which may limit the generalizability of our findings. Readers should bear in mind that the purpose of this retrospective, non-pre-specified study was to explore and compare the prognostic potential of existing approaches, not to develop and validate new prediction models. Thus, our findings should only be considered hypothesis-generating.

## Conclusion

We found that the SQ, the NoH, and the combination of both could help identify advanced HF patients at increased risk of 1-year mortality; these simple prognostication tools might have the potential to support decision making around advanced HF therapies, inform conversations around goals of care, and raise awareness for unmet palliative care needs.

## Data Availability Statement

The original contributions presented in the study are included in the article/supplementary material, further inquiries can be directed to the corresponding author.

## Ethics Statement

The studies involving human participants were reviewed and approved by the Program for the Protection of Human Subjects (PPHS) at the Icahn School of Medicine at Mount Sinai. The patients/participants provided their written informed consent to participate in this study.

## Author Contributions

MB, LG, and NG contributed to conception and design of the study. LG and NG were responsible for investigation, funding acquisition, project administration, and supervision. KM contributed to methodology, data curation, and statistical analysis. MB wrote the first draft and submitted the manuscript. KM, LG, NG, and SP reviewed and edited the manuscript. All authors contributed to the article and approved the submitted version.

## Conflict of Interest

SP is a consultant to Cordio Medical Ltd., and has received consulting fees from Abbott, CareDx, Medtronic, NuPulse, Procyrion, and Transmedics. The remaining authors declare that the research was conducted in the absence of any commercial or financial relationships that could be construed as a potential conflict of interest.

## Publisher’s Note

All claims expressed in this article are solely those of the authors and do not necessarily represent those of their affiliated organizations, or those of the publisher, the editors and the reviewers. Any product that may be evaluated in this article, or claim that may be made by its manufacturer, is not guaranteed or endorsed by the publisher.

## References

[1] Crespo-Leiro MG Metra M Lund LH Milicic D Costanzo MR Filippatos G Advanced heart failure: a position statement of the Heart Failure Association of the European Society of Cardiology. *Eur J Heart Fail.* (2018) 20:1505–35. 10.1002/ejhf.1236 29806100

[2] McDonagh TA Metra M Adamo M Gardner RS Baumbach A Böhm M 2021 ESC Guidelines for the diagnosis and treatment of acute and chronic heart failure. *Eur Heart J.* (2021) 42:3599–726. 34447992 10.1093/eurheartj/ehab368

[3] Kavalieratos D Gelfman LP Tycon LE Riegel B Bekelman DB Ikejiani DZ Palliative care in heart failure: rationale, evidence, and future priorities. *J Am Coll Cardiol.* (2017) 70:1919–30. 10.1016/j.jacc.2017.08.036 28982506 PMC5731659

[4] Rogers JG Patel CB Mentz RJ Granger BB Steinhauser KE Fiuzat M Palliative care in heart failure: the PAL-HF randomized, controlled clinical trial. *J Am Coll Cardiol.* (2017) 70:331–41. 10.1016/j.jacc.2017.05.030 28705314 PMC5664956

[5] O’Donnell AE Schaefer KG Stevenson LW DeVoe K Walsh K Mehra MR Social worker-aided palliative care intervention in high-risk patients with heart failure (SWAP-HF): a pilot randomized clinical trial. *JAMA Cardiol.* (2018) 3:516–9. 10.1001/jamacardio.2018.0589 29641819 PMC6128511

[6] Bakitas M MacMartin M Trzepkowski K Robert A Jackson L Brown J Palliative care consultations for heart failure patients: how many, when, and why? *J Card Fail.* (2013) 19:193–201. 10.1016/j.cardfail.2013.01.011 23482081 PMC4564059

[7] Glare PA Sinclair CT. Palliative medicine review: prognostication. *J Palliat Med.* (2008) 11:84–103. 10.1089/jpm.2008.9992 18370898

[8] Chu C White N Stone P. Prognostication in palliative care. *Clin Med.* (2019) 19:306–10. 10.7861/clinmedicine.19-4-306 PMC6752241 31308109

[9] Straw S Byrom R Gierula J Paton MF Koshy A Cubbon R Predicting one-year mortality in heart failure using the ‘Surprise Question’: a prospective pilot study. *Eur J Heart Fail.* (2019) 21:227–34. 10.1002/ejhf.1353 30548129

[10] Gonzalez-Jaramillo V Ochoa LFA Saldarriaga C Krikorian A Vargas JJ Gonzalez-Jaramillo N The ‘Surprise question’ in heart failure: a prospective cohort study. *BMJ Support Palliat Care.* (2021): [Online ahead of print], 10.1136/bmjspcare-2021-003143 34404746 PMC10894837

[11] Setoguchi S Stevenson LW Schneeweiss S. Repeated hospitalizations predict mortality in the community population with heart failure. *Am Heart J.* (2007) 154:260–6. 10.1016/j.ahj.2007.01.041 17643574

[12] Goldstein NE Mather H McKendrick K Gelfman LP Hutchinson MD Lampert R Improving communication in heart failure patient care. *J Am Coll Cardiol.* (2019) 74:1682–92. 10.1016/j.jacc.2019.07.058 31558252 PMC7000126

[13] Goldstein NE Kalman J Kutner JS Fromme EK Hutchinson MD Lipman HI A study to improve communication between clinicians and patients with advanced heart failure: methods and challenges behind the working to improve discussions about defibrillator management trial. *J Pain Symptom Manage.* (2014) 48:1236–46. 10.1016/j.jpainsymman.2014.03.005 24768595 PMC4205212

[14] Levy WC Mozaffarian D Linker DT Sutradhar SC Anker SD Cropp AB The seattle heart failure model: prediction of survival in heart failure. *Circulation.* (2006) 113:1424–33. 10.1161/CIRCULATIONAHA.105.584102 16534009

[15] Youden WJ. Index for rating diagnostic tests. *Cancer.* (1950) 3:32–5. 10.1002/1097-0142(1950)3:1<32::aid-cncr2820030106>3.0.co;2-3 15405679

[16] Kalogeropoulos AP Georgiopoulou VV Giamouzis G Smith AL Agha SA Waheed S Utility of the Seattle Heart Failure Model in patients with advanced heart failure. *J Am Coll Cardiol.* (2009) 53:334–42. 10.1016/j.jacc.2008.10.023 19161882

[17] Gorodeski EZ Chu EC Chow CH Levy WC Hsich E Starling RC. Application of the Seattle Heart Failure Model in ambulatory patients presented to an advanced heart failure therapeutics committee. *Circ Heart Fail.* (2010) 3:706–14. 10.1161/CIRCHEARTFAILURE.110.944280 20798278

[18] Lanfear DE Levy WC Stehlik J Estep JD Rogers JG Shah KB Accuracy of seattle heart failure model and HeartMate II risk score in non-inotrope-dependent advanced heart failure patients: insights from the ROADMAP Study (Risk assessment and comparative effectiveness of left ventricular assist device and medical management in ambulatory heart failure patients). *Circ Heart Fail.* (2017) 10:e003745. 10.1161/CIRCHEARTFAILURE.116.003745 28465311

[19] Aaronson EL George N Ouchi K Zheng H Bowman J Monette D The surprise question can be used to identify heart failure patients in the emergency department who would benefit from palliative care. *J Pain Symptom Manage.* (2019) 57:944–51. 10.1016/j.jpainsymman.2019.02.007 30776539 PMC6713219

[20] University of Washington. *Seattle Heart Failure Model.* (2017). Available online at:https://depts.washington.edu/shfm (accessed February 24, 2022).

[21] Murray S Boyd K. Using the ‘surprise question’ can identify people with advanced heart failure and COPD who would benefit from a palliative care approach. *Palliat Med.* (2011) 25:382. 10.1177/0269216311401949 21610113

[22] Chang YK Kaplan H Geng Y Mo L Philip J Collins A Referral criteria to palliative care for patients with heart failure: a systematic review. *Circ Heart Fail.* (2020) 13:e006881. 10.1161/CIRCHEARTFAILURE.120.006881 32900233 PMC7494548

[23] Gelfman LP Mather H McKendrick K Wong AY Hutchinson MD Lampert RJ Non-concordance between patient and clinician estimates of prognosis in advanced heart failure. *J Card Fail.* (2021) 27:700–5. 10.1016/j.cardfail.2021.03.005 34088381 PMC8186811

[24] Gelfman LP Sudore RL Mather H McKendrick K Hutchinson MD Lampert RJ Prognostic awareness and goals of care discussions among patients with advanced heart failure. *Circ Heart Fail.* (2020) 13:e006502. 10.1161/CIRCHEARTFAILURE.119.006502 32873058 PMC7494630

[25] Warraich HJ Xu H DeVore AD Matsouaka R Heidenreich PA Bhatt DL Trends in hospice discharge and relative outcomes among medicare patients in the get with the guidelines-heart failure registry. *JAMA Cardiol.* (2018) 3:917–26. 10.1001/jamacardio.2018.2678 30167645 PMC6233829

[26] Greener DT Quill T Amir O Szydlowski J Gramling RE. Palliative care referral among patients hospitalized with advanced heart failure. *J Palliat Med.* (2014) 17:1115–20. 10.1089/jpm.2013.0658 25068475

[27] Bozkurt B Coats AJS Tsutsui H Abdelhamid CM Adamopoulos S Albert N Universal definition and classification of heart failure: a report of the Heart Failure Society of America, Heart Failure Association of the European Society of Cardiology, Japanese Heart Failure Society and Writing Committee of the Universal Definition of Heart Failure: endorsed by the Canadian Heart Failure Society, Heart Failure Association of India, Cardiac Society of Australia and New Zealand, and Chinese Heart Failure Association. *Eur J Heart Fail.* (2021) 23:352–80. 10.1002/ejhf.2115 33605000

